# Asialoglycoprotein receptor-magnetic dual targeting nanoparticles for delivery of RASSF1A to hepatocellular carcinoma

**DOI:** 10.1038/srep22149

**Published:** 2016-02-26

**Authors:** Wan-Jiang Xue, Ying Feng, Fei Wang, Yi-Bing Guo, Peng Li, Lei Wang, Yi-Fei Liu, Zhi-Wei Wang, Yu-Min Yang, Qin-Sheng Mao

**Affiliations:** 1 Department of General Surgery, Nantong University Affiliated Hospital, Nantong 226001, Jiangsu, China; 2 Surgical Comprehensive Laboratory, Nantong University Affiliated Hospital, Nantong 226001, Jiangsu, China; 3 Department of Pathology, Nantong University Affiliated Hospital, Nantong 226001, Jiangsu, China; 4 Jiangsu Key Laboratory of Neuroregeneration, Nantong University, Nantong 226007, Jiangsu, China; 5 The Neural Regeneration Co-innovation Center of Jiangsu Province, Nantong University, Nantong 226007, Jiangsu, China

## Abstract

We developed a nanovector with double targeting properties for efficiently delivering the tumor suppressor gene RASSF1A specifically into hepatocellular carcinoma (HCC) cells by preparing galactosylated-carboxymethyl chitosan-magnetic iron oxide nanoparticles (Gal-CMCS-Fe_3_O_4_-NPs). After conjugating galactose and CMCS to the surface of Fe_3_O_4_-NPs, we observed that Gal-CMCS-Fe_3_O_4_-NPs were round with a relatively stable zeta potential of +6.5 mV and an mean hydrodynamic size of 40.1 ± 5.3 nm. Gal-CMCS-Fe_3_O_4_-NPs had strong DNA condensing power in pH 7 solution and were largely nontoxic. *In vitro* experiments demonstrated that Gal-CMCS-Fe_3_O_4_-NPs were highly selective for HCC cells and liver cells. *In vivo* experiments showed the specific accumulation of Gal-CMCS-Fe_3_O_4_-NPs in HCC tissue, especially with the aid of an external magnetic field. Nude mice with orthotopically transplanted HCC received an intravenous injection of the Gal-CMCS-Fe_3_O_4_-NPs/pcDNA3.1(+)RASSF1A compound and intraperitoneal injection of mitomycin and had an external magnetic field applied to the tumor area. These mice had the smallest tumors, largest percentage of TUNEL-positive cells, and highest caspase-3 expression levels in tumor tissue compared to other groups of treated mice. These results suggest the potential application of Gal-CMCS-Fe_3_O_4_-NPs for RASSF1A gene delivery for the treatment of HCC.

Hepatocellular carcinoma (HCC) is a major malignant disease and a threat to global public health^1^. Of the approximately 782, 500 new cases reported annually, nearly half occur in China^2^. Currently, combined therapies, including surgery, radiofrequency ablation, transcatheter hepatic arterial chemoembolization, biotherapy, targeted drug therapy, traditional Chinese medicine, and liver transplantation, are used to treat HCC^3^,^4^. However, most HCC patients, who often have liver cirrhosis and chronic hepatitis B infection, have a low tolerance for treatment, poor rates of surgical resection, a tendency for postoperative HCC recurrence and metastasis, and unsatisfactory chemotherapy results. Therefore, new treatments such as gene therapy may be effective supplements to conventional treatments^5^.

The success of gene therapy depends on the ability to identify suitable targets. Studies show that Ras Association Domain Family 1A (RASSF1A) is a tumor suppressor gene that is implicated in the ras signaling pathway and has been shown to play a critical role in apoptosis, cell-cycle regulation, and microtubule stability^6^,^7^. Numerous tumor tissues including HCC are lacking RASSF1A expression due to hypermethylation of its promoter region^8^,^9^,^10^. This hypermethylation is associated with distant metastasis and low survival rates of patients after tumorectomy, making it a molecular marker for predicting HCC prognosis^11^,^12^. Re-expression of the RASSF1A gene not only inhibits the growth of HCC cells *in vitro* and *in vivo* but also increases the sensitivity of HCC cells to the chemotherapy drug mitomycin (MMC)^13^. Therefore, restoring the function of RASSF1A in HCC tissue could be a strategy for HCC gene therapy.

The use of nanoparticles (NPs) as a vector for gene therapy has attracted much attention^14^. Fe_3_O_4_-NPs are one of the most widely utilized magnetic particles^15^. With a diameter of less than 30 nm, Fe_3_O_4_-NPs have the characteristic of superparamagnetism, which enables their movement and concentration in the body to be controlled with an external magnetic field and allows NPs to be used as carriers of gene medicine or RNA^16^. Coating magnetic Fe_3_O_4_-NPs with proteins, liposomes, polysaccharides, and other bio-macromolecules improves their biocompatibility^17^,^18^. In particular, chitosan is a polysaccharide that is generally accepted as a safe and nontoxic biomaterial and DNA vector^19^. Chitosan can be combined with magnetic particles to form magnetic microspheres that directly couple with specific ligands and can easily be used to modify NPs surfaces^20^. Furthermore, the naturally hydrophilic surface of chitosan can abate phagocytosis by macrophages in the body, thus prolonging its circulation in the blood^21^.

Difficulty in targeting vectors to the liver and low transfection efficiencies are major obstacles for HCC gene therapy^22^. Recently, attention has focused on modifying the surfaces of NPs with specific ligands, thus targeting them to the liver via receptor-mediated pathways after intravenous administration^23^. Asialoglycoprotein receptors (ASGP-Rs), with a density of approximately 500,000 receptors per cell^24^, are an important target of hepatocyte-targeted delivery systems^25^. However, ASGP-R expression is reduced in HCC, especially in Edmondson Grade III-IV HCC, which may result in inefficient gene delivery^26^. Therefore, alterations in the physicochemical properties of NPs are still needed to produce ideal vectors for the delivery of therapeutic genes to HCC tissue.

In this study, we used carboxymethyl chitosan (CMCS) and magnetic Fe_3_O_4_ to prepare galactose-CMCS-Fe_3_O_4_-NPs (Gal-CMCS-Fe_3_O_4_-NPs) by taking the free amino groups of CMCS molecules as cross-linked groups and coupling them with galactose (Gal) ligands using the ammoniation reduction method. We took advantage of the superior biocompatibility and biodegradability of CMCS compared with chitosan^27^, the ability to specifically target galactose to liver cells, and the magnetic targeting capability of Fe_3_O_4_-NPs to create a new efficient gene vector with double targeting properties for HCC.

## Results

### Physical and chemical analysis of Gal-CMCS-Fe_3_O_4_-NPs

**Figure 1:**
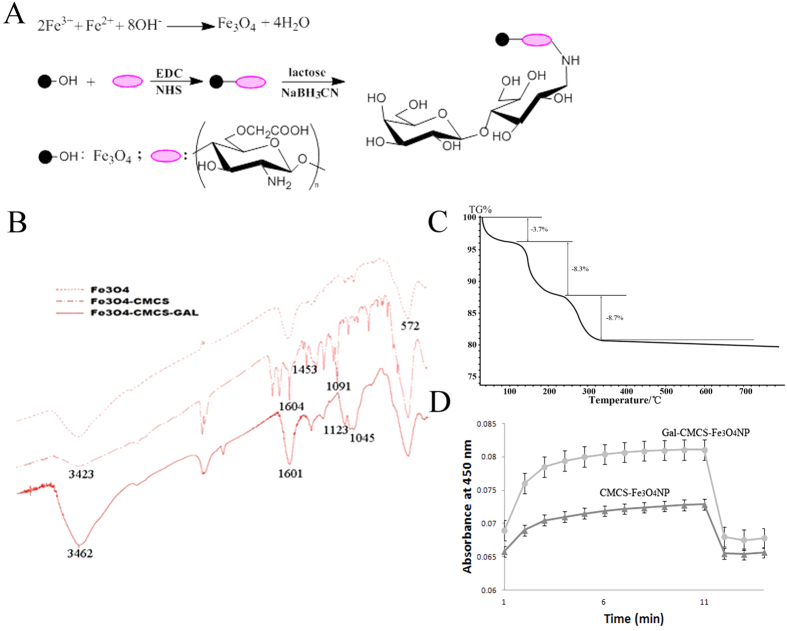
Identification of Gal-CMCS-Fe_3_O_4_-NPs. (**A**) Synthesis of Gal-CMCS-Fe_3_O_4_-NPs. (**B**) Infrared spectrum analysis of Gal-CMCS-Fe_3_O_4_-NPs. (**C**) Thermogravimetric analysis of Gal-CMCS-Fe_3_O_4_-NPs. (**D**) RCA120-induced aggregation of Gal-CMCS-Fe_3_O_4_-NPs and CMCS-Fe_3_O_4_-NPs.

Figure 1A depicts the synthesis of Gal-CMCS-Fe_3_O_4_-NPs. Infrared spectrum analysis revealed that the primary absorption peak of Fe_3_O_4_-NPs was attributed to the vibration of Fe-O. The series of absorption peaks of CMCS-Fe_3_O_4_-NPs included the stretch vibration absorption peak of -NH_2_ and -OH at 3423 cm^−1^, the anti-symmetric vibration peak of -COO and the symmetric vibration absorption peak of -COO- at 1604 cm^−1^ and 1453 cm^−1^, respectively, and the shoulder peaks of the sugar ring at 1091 cm^−1^ and 1219 cm^−1^. The strengthening and widening of the absorption peak at 1601 cm^−1^ in Gal-CMCS-Fe_3_O_4_-NPs indicated that -NH_2_ had linked with related groups. The stretching vibration of the C-N key at 1123 cm^−1^ also indicated the introduction of galactosyl, whereas the widening of peaks at 3462 cm^−1^ and 1045 cm^−1^ indicated that the introduction of galactosyl resulted in an increase in hydroxyl (Fig. 1B). The thermal gravimetric curve for Gal-CMCS-Fe_3_O_4_-NPs showed three weight loss events as follows: the first from the room temperature to 100 °C, which may be due to evaporation of water on the surface of Gal-CMCS-Fe_3_O_4_-NPs, the second from 100° to 200 °C, which can be attributed to the decomposition of galactose, and the third from 200° to 350 °C (Fig. 1C), which can be attributed to the decomposition of CMCS.

To evaluate the binding of galactose moieties on Gal-CMCS-Fe_3_O_4_-NPs to galactose-recognizing lectins, the aggregation of Gal-CMCS-Fe_3_O_4_-NPs induced by ricinus communis agglutinin I (RCA120) was measured by changes in turbidity over time. When RCA120 was added, Gal-CMCS-Fe_3_O_4_-NPs had a greater absorbance than CMCS-Fe_3_O_4_-NPs. Consistent with characteristics of RCA120^28^, when excess of a galactose competitive antagonist was added, Gal-CMCS-Fe_3_O_4_-NPs decomposed, and the absorbencies of Gal-CMCS-Fe_3_O_4_-NPs and CMCS-Fe_3_O_4_-NPs became similar(Fig. 1D).

**Figure 2:**
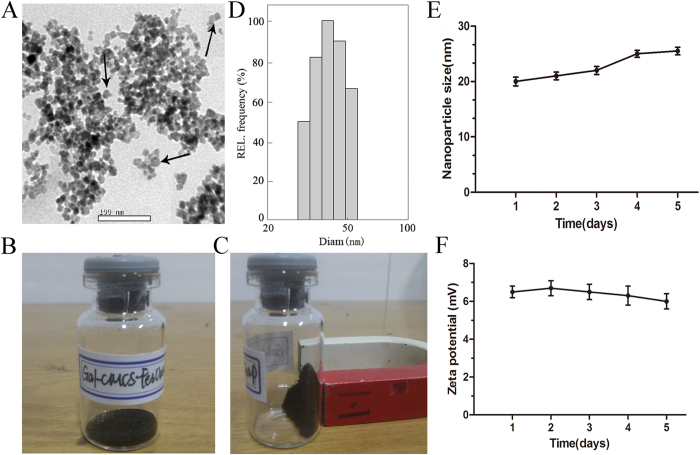
Characterization of Gal-CMCS-Fe_3_O_4_-NPs. (**A**) TEM image of Gal-CMCS-Fe_3_O_4_-NPs. (**B,C**) Magnetic performance of Gal-CMCS-Fe_3_O_4_-NPs. (**D**) hydrodynamic size distribution of Gal-CMCS-Fe_3_O_4_-NPs. (**E**) Stability of hydrodynamic size of Gal-CMCS-Fe_3_O_4_-NPs over time. (**F**) Stability of zeta potential of Gal-CMCS-Fe_3_O_4_-NPs over time.

Transmission electron microscope (TEM) showed that Gal-CMCS-Fe_3_O_4_-NPs had a relatively uniform round shape (Fig. 2A). The average primary particle diameter of Gal-CMCS-Fe_3_O_4_-NPs was 20.0 ± 2.5 nm. Use of a magnet showed that Gal-CMCS-Fe_3_O_4_-NPs had good magnetic responsiveness (Fig. 2B,C). The saturation magnetization for Gal-CMCS-Fe_3_O_4_-NPs at room temperature was 38.23 emu/g using Lake Shore 7407 vibrating sample magnetometer. The mean zeta potential and hydrodynamic size of Gal-CMCS- Fe_3_O_4_-NPs in water were measured using Nicomp 380 ZLS and were found to be +6.5 mV and 40.1 ± 5.3 nm (Fig. 2D), respectively. At pH 7, the hydrodynamic size (Fig. 2E) and zeta potential (Fig. 2F) of Gal-CMCS- Fe_3_O_4_-NPs were relatively stable across 5 days of observation in water. There was no statistically significant difference between water and Dulbecco’s modified Eagle’s medium (DMEM) with relevant to hydrodynamic size and zeta potential of Gal-CMCS-Fe_3_O_4_-NPs (P > 0.05).

### Hemolysis assay and toxicity assessment

**Figure 3:**
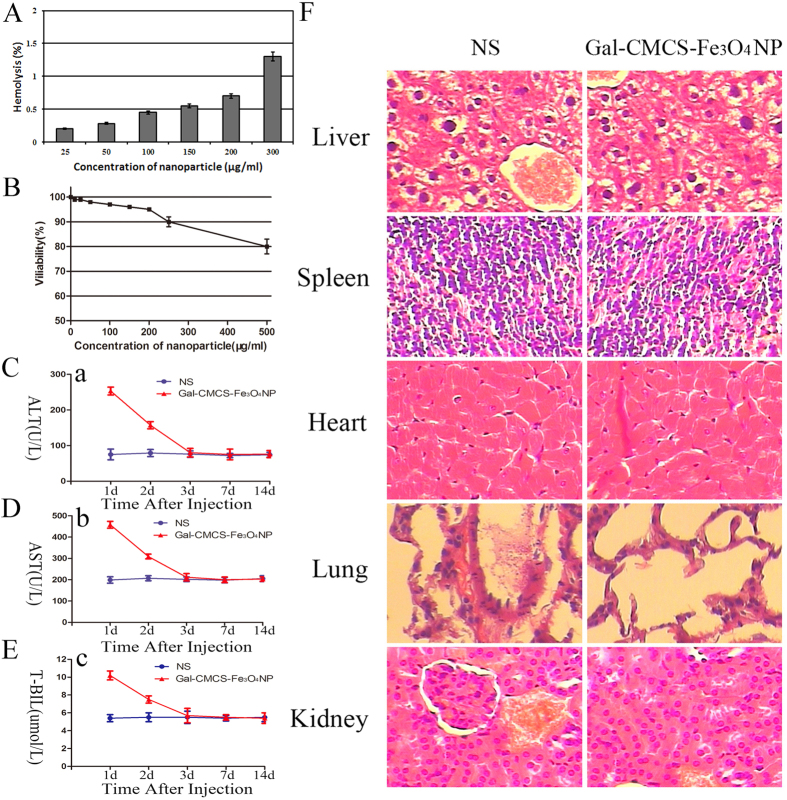
Hemolysis assay and toxicity tests of Gal-CMCS-Fe_3_O_4_-NPs *in vitro* and *in vivo*. (**A**) Hemolysis of the Gal-CMCS-Fe_3_O_4_-NPs at various concentrations. (**B**) Toxicity test of Gal-CMCS-Fe_3_O_4_-NPs using L02 cells. (**C–E**) Effect of Gal-CMCS-Fe_3_O_4_-NPs injection on mouse liver function. (**F**) Effect of Gal-CMCS-Fe_3_O_4_-NPs injection on morphology of mouse organs.

Hemolysis of the Gal-CMCS-Fe_3_O_4_-NPs was investigated for its hemocompatibility as shown in Fig. 3A. The degree of hemolysis of all the tested Gal-CMCS-Fe_3_O_4_-NP samples at different concentrations were below 2%.

An *in vitro* toxicity test showed that when exposed to a concentration of 200 μg/ml Gal-CMCS-Fe_3_O_4_-NPs, the viability of L02 cells was over 95% (Fig. 3B). As Gal-CMCS-Fe_3_O_4_-NP concentration approached 500 μg/ml, cell viability decreased but remained high at 80%. Therefore, a concentration of 200 μg/ml Gal-CMCS-Fe_3_O_4_-NPs was chosen for subsequent experiments.

Gal-CMCS-Fe_3_O_4_-NPs were injected into the tail vein of nude mice and serum was collected to assess liver function 1, 2, 3, 7, or 14 days later. Gal-CMCS-Fe_3_O_4_-NPs induced transient toxicity, as ALT, AST, and T-BIL levels were higher than in the control group on day 1 and 2 (*P* < 0.05) but returned to normal by day 3 (*P* > 0.05; Fig. 3C–E). After Gal-CMCS-Fe_3_O_4_-NPs injection, mice showed no signs of acute toxic reaction, discomfort, or fatigue and slowly gained weight over the 14-day observation period. After hematoxylin and eosin staining of paraffin-embedded sections, optical microscopy revealed no obvious differences in the morphology of primary organs between the normal saline (NS) and Gal-CMCS-Fe_3_O_4_-NPs groups (Fig. 3F).

### Characterization of Gal-CMCS-Fe_3_O_4_-NPs/DNA complexes

**Figure 4:**
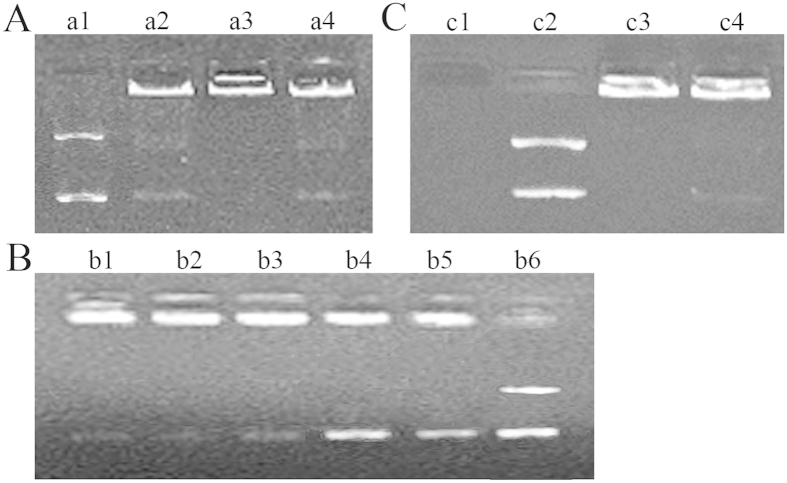
Gel retardation analysis of Gal-CMCS-Fe_3_O_4_-NPs/DNA complexes. (**A**) Effect of different pH levels on Gal-CMCS-Fe_3_O_4_-NPs/DNA binding (lane a1: pH = 7, only plasmid; lane a2-a4: NPs/DNA mass ratio of 3:1, pH = 5, 7, and 9, respectively). (**B**) Effect of different mass ratios on Gal-CMCS-Fe_3_O_4_-NPs/DNA binding (lanes b1-b5: NPs/DNA mass ratio of 4:1, 3:1, 2:1, 1:1, and 0.5:1, respectively; lane b6: plasmid only). (**C**) DNA protection by Gal-CMCS-Fe_3_O_4_-NPs (lane c1: plasmid + DNase I; lane c2: only plasmid; lane c3: Gal-CMCS-Fe_3_O_4_-NPs/DNA + DNase I; lane c4: Gal-CMCS-Fe_3_O_4_-NPs/DNA).

Under a pH of 5, 7, or 9, the swimming speed of Gal-CMCS-Fe_3_O_4_-NP/DNA was lower than that of the plasmid-only group (Fig. 4A). In addition, a small amount of free DNA was observed under the three different pH conditions. However, the lowest level of free DNA was associated with a pH of 7 (close to the pH of the human body). At a pH of 7, as the concentration of Gal-CMCS-Fe_3_O_4_-NPs increased, the retention of DNA also increased (Fig. 4B). When the NPs/DNA mass ratio was 3:1, Gal-CMCS-Fe_3_O_4_-NPs retained all DNA. Therefore, a mass ratio of 3:1 was used in subsequent experiments. Electrophoresis of Gal-CMCS-Fe_3_O_4_-NPs/DNA after treatment with the digestive enzyme DNase I showed that DNA coated with NPs had no obvious fragments, whereas DNA fragments were observed with uncoated DNA (Fig. 4C).

### Targeted transfection of HCC cells with Gal-CMCS-Fe_3_O_4_-NPs *in vitro*

**Figure 5:**
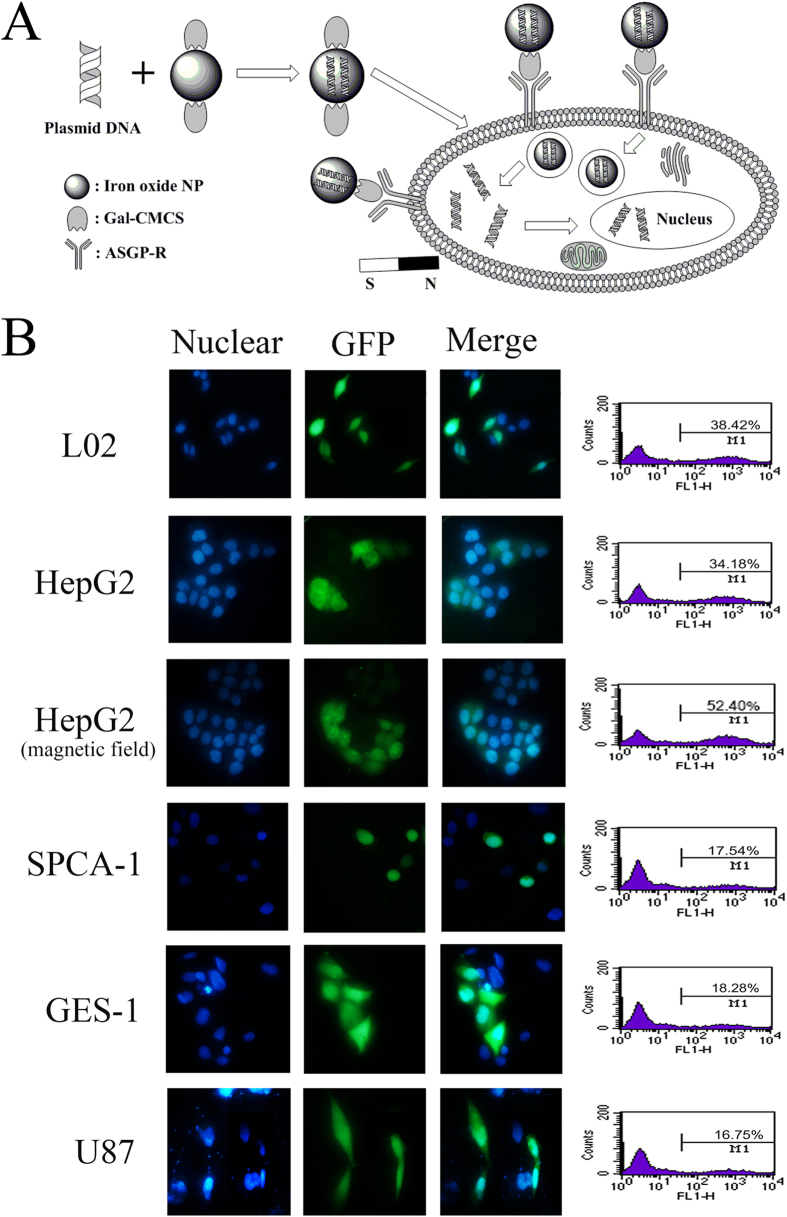
Targeted transfection of HCC cells with Gal-CMCS-Fe_3_O_4_-NPs *in vitro*. (**A**) Schematic diagram of the entry of Gal-CMCS-Fe_3_O_4_-NPs inside the nucleus of cell. (**B**) Transfection efficiency of Gal-CMCS-Fe_3_O_4-_NPs/pcDNA6.2mir-EGFP in different cell lines. M1 = the percentage of transfected cells with green fluorescence.

**Figure 6:**
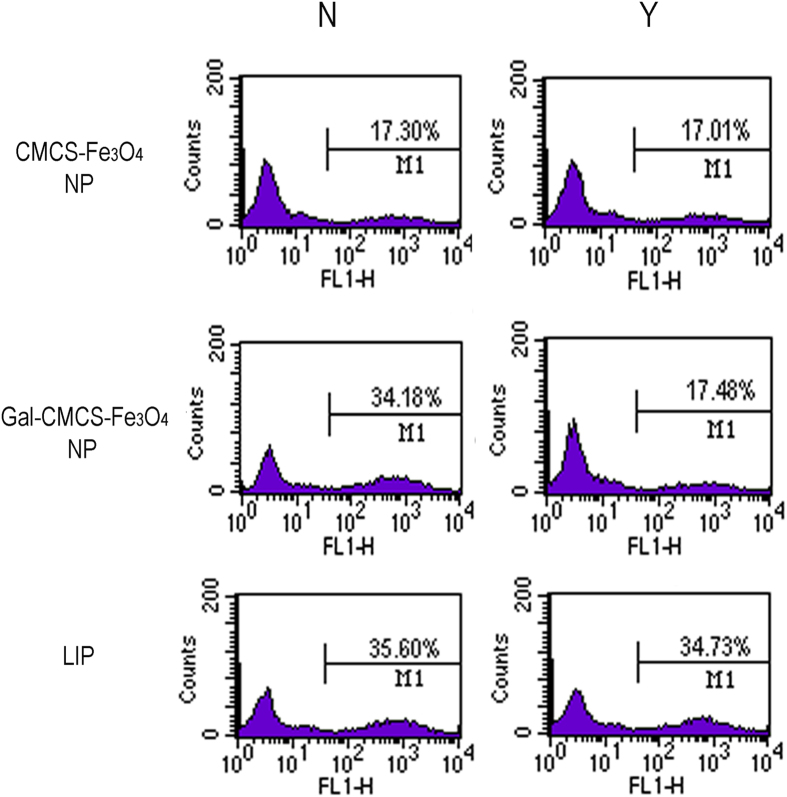
Effect of galactose on the transfection efficiency of Gal-CMCS-Fe_3_O_4_NPs/pcDNA6.2mir-EGFP in HepG2 cells. N = no addition of galactose; Y = addition of galactose; LIP = Lipofectamine 2000, M1 = the percentage of transfected cells with green fluorescence.

Figure 5A depicts the schematic diagram of the entry of Gal-CMCS-Fe_3_O_4_-NPs inside the nucleus of cell. To investigate the targeting specificity of Gal-CMCS-Fe_3_O_4_-NPs for HCC cells, NPs were used to transfect plasmids into HepG2, L02, GES-1, U87, and SPCA-1 cell lines (Fig. 5B). Seventy-two hours after transfection, strong green fluorescence was observed in liver cells (L02 and HepG2), whereas weaker fluorescence was observed in non-liver cells (GES-1, U87 and SPCA-1). Flow cytometry showed that the average transfection efficiency of pcDNA6.2mir-EGFP in L02 and HepG2 cells was 39.12 ± 2.56% and 35.23 ± 2.33%, respectively (*P* > 0.05), whereas transfection efficiency in SPCA-1, GES-1, and U87 cells was only 18.01 ± 1.97%, 18.89 ± 1.86%, and 16.99 ± 1.64%, respectively. This difference in Gal-CMCS-Fe_3_O_4_-NPs transfection efficiency between liver and non-liver cells was statistically significant (*P* < 0.01). Additionally, approximately 54.55 ± 4.27% of HepG2 cells were transfected with the aid of an external magnetic field, whereas 35.23 ± 2.33% of HepG2 cells were transfected without an external magnetic field (*P* < 0.05). The addition of galactose decreased the transfection efficiency of Gal-CMCS-Fe_3_O_4_-NPs in HepG2 cells from 35.23 ± 2.33% to 18.93 ± 1.96% (*P* < 0.05; Fig. 6). However, the transfection efficiency of CMCS-Fe_3_O_4_-NPs was similar with or without the addition of galactose (*P* > 0.05; Fig. 6).

### Targeted transfection of HCC tissue by Gal-CMCS-Fe_3_O_4_-NPs *in vivo*

**Figure 7:**
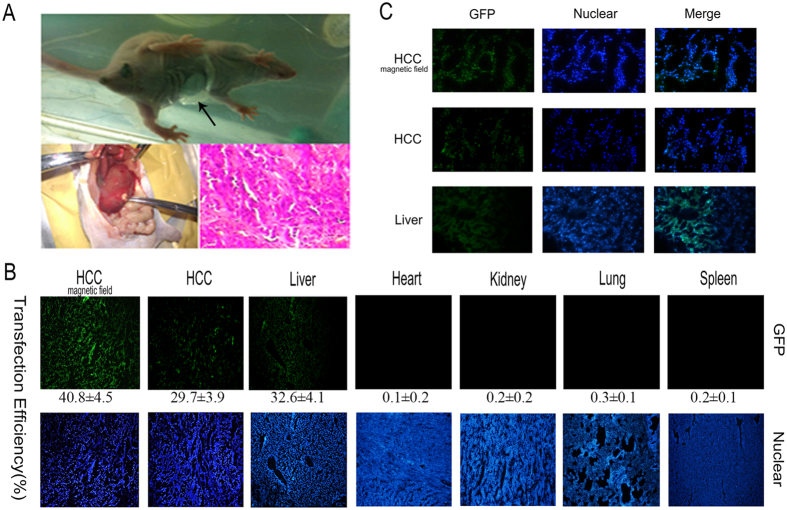
Transfection efficiency of Gal-CMCS-Fe_3_O_4_-NPs/pcDNA6.2 mir-EGFP in different mouse organs. (**A**) Orthotopic transplantation of HCC in mice. The arrow marks the position of the small magnet. (**B**) Transfection efficiency of Gal-CMCS-Fe_3_O_4_-NPs/pcDNA6.2mir-EGFP in different mouse organs. (**C**) Transfection of Gal-CMCS-Fe_3_O_4_-NPs/pcDNA6.2mir-EGFP in liver and orthotopically transplanted HCC tissue.

After removing subcutaneous tumors composed of HepG2 cells from nude mice, orthotopic transplantation of the tumors under capsula fibrosa was performed. Two weeks later, Gal-CMCS-Fe_3_O_4_-NP/pcDNA6.2mir-EGFP compound was injected into the tail vein of mice. After 3 days, mice were sacrificed and livers, kidneys, spleen, heart, lungs, and orthotopically transplanted HCC tissue were removed (Fig. 7A). We observed green fluorescence in liver and HCC tissue sections. The average transfection efficiency of pcDNA6.2mir-EGFP in liver tissue was 32.6%, Furthermore, the average transfection efficiency was approximately 40.8% in HCC tissue with the aid of an external magnetic field, and 29.7% in HCC tissue without an external magnetic field (*P* < 0.01; Fig. 7B,C). No obvious fluorescence was observed in kidney, spleen, heart, or lung tissue sections (Fig. 7B).

### Efficient delivery of the RASSF1A gene for HCC treatment by Gal-CMCS-Fe_3_O_4_-NPs combined with MMC

**Figure 8:**
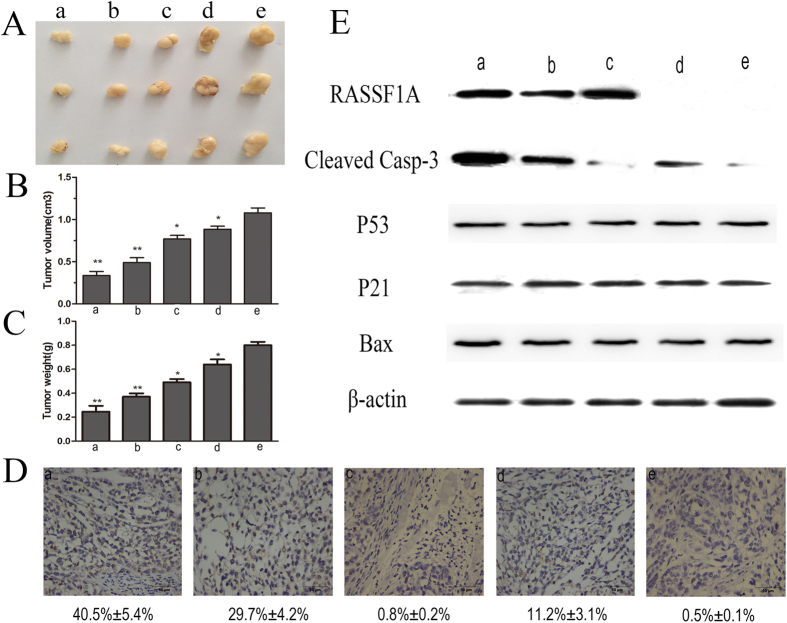
Therapeutic effect of Gal-CMCS-Fe_3_O_4_-NPs/RASSF1A combined with MMC on HCC in nude mice. (**A**) Tumors from mice treated with Gal-CMCS-Fe_3_O_4_-NPs/pcDNA3.1(+)RASSF1A + MMC + magnetic field (group a), Gal-CMCS-Fe_3_O_4_NPs/pcDNA3.1(+)RASSF1A + MMC (group b), Gal-CMCS-Fe_3_O_4_NPs/pcDNA3.1(+)RASSF1A + saline + magnetic field (group c), Gal-CMCS-Fe_3_O_4_NPs/pcDNA3.1(+) + MMC + magnetic field (group d), Gal-CMCS-Fe_3_O_4_NPs/pcDNA3.1(+) + saline + magnetic field (group e). (**B**) Tumor volume. (**C**) Tumor weight. (**D**) TUNEL assay. (**E**) Apoptosis-related protein expression detected by western blot. *P < 0.05, **P < 0.001.

Two weeks after the orthotopic HCC transplantation model mice received treatment, tumor volumes and weights were lower in treated mice than in control mice (group a, *P* < 0.01; group b, *P* < 0.01; group c, *P* < 0.05; group d, *P* < 0.05; Fig. 8A–C). Among the treatment groups, intravenous injection of RASSF1A-NPs and intraperitoneal injection of MMC with the aid of an external magnetic field (group a) inhibited tumor growth the most. The average percent of terminal deoxynucleotidyl transferase-mediated dUTP nick end labeling (TUNEL) positive cells in the four treatment groups and control group were 40.5%, 29.7%, 0.8%, 11.2%, and 0.5%, respectively (Fig. 8D). RASSF1A expression in tumor tissue was higher in groups a and c than in group b, whereas RASSF1A expression was not observed in group d or in the control group (group e; Fig. 8E). Caspase-3 expression in tumor tissue was higher in groups a, b, and d than in group c or the control group. There were no differences between groups in p53, p21, bcl-2, or bax expression.

## Discussion

The purpose of targeted gene therapy is to assemble the desired genes with a suitable carrier that can target specific tissues and enable effective gene expression^29^. The design and development of NPs with high transfection efficiency and low cytotoxicity are critical for successful gene therapy^30^. Super paramagetic iron oxide NPs targeted to specific cells for magnetic resonance imaging, tissue repair, targeted drug delivery, and hyperthermia with a large number of polycations, including chitosan, polyethylenamine, polyamidoamine, and polyamines have been receiving considerable attention^31^. It is necessary to introduce functional ligands such as galactose, folic acid, epithelial cell adhesion molecule, and α-fetoprotein that can actively interact with the corresponding binding sites on the cell surfaces of HCC to further improve the binding of ligands to specific receptor targets^32^. However, the common challenge among these applications is to ensure sufficient uptake of NPs by HCC cells. Furthermore, the potential toxic effects of these NPs *in vivo* also remain unclear^33^.

Here, with the aim of enhancing targeted HCC gene therapy, we constructed Gal-CMCS-Fe_3_O_4_-NPs that could be used for transfection *in vivo* and *in vitro*, were safe and efficient, and could be used with an external magnetic field to target the liver. Examination with a laser particle size analyzer showed that vector particles had a diameter of approximately 40.1 nm, which is beneficial for a HCC-targeted gene carrier^34^,^35^. ASGP-R-mediated endocytosis of galactose-modified delivery systems is influenced by the size of NPs^36^, with NPs less than 50 nm in diameter efficiently targeting hepatocytes and NPs over 140 nm in diameter being more selective for Kupffer cells. Therefore, the Gal-CMCS-Fe_3_O_4_-NPs prepared in this study should be absorbed by HCC cells^37^.

We further investigated the chemical and structural properties of Gal-CMCS- Fe_3_O_4_-NPs using infrared spectrum and thermogravimetric analyses. Infrared spectrum analysis of CMCS-Fe_3_O_4_-NPs revealed bands at 3423cm^−1^ (-NH2, -OH), 1604 cm^−1^ and 1453 cm^−1^ (vas, vs, -COO-), 1091 cm^−1^, and 1219 cm^−1^, indicating the introduction of glycosyl (Fig. 2A). Gal-CMCS-Fe_3_O_4_-NPs exhibited bands at 3462 cm^−1^, 1601 cm^−1^ (-NH_2_), 1123 cm^−1^ (C-N), and 1045 cm^−1^ (-OH), indicating the introduction of galactosyl. The thermogravimetric curve indicated two major weight loss events: one at 100–200 °C, the other at 200–350 °C. The percentage of CMCS and Gal on Gal-CMCS-Fe_3_O_4_-NPs was determined as 8.7% and. 8.3% (m/m), respectively. These results provide evidence of the successful preparation of Gal-CMCS-Fe_3_O_4_-NPs. An RCA120 test showed that Gal-CMCS-Fe_3_O_4_-NPs exhibited greater absorbance than did CMCS-Fe_3_O_4_-NPs, and the addition of excess galactose decreased absorbance, indicating the galactose-specific binding of Gal-CMCS-Fe_3_O_4_-NPs with RCA120, which indirectly shows that the surface of Gal-CMCS-Fe_3_O_4_-NPs was coated with galactosyl.

The hydrodynamic size of Gal-CMCS-Fe_3_O_4_-NPs is composed of three parts namely Fe_3_O_4_-NPs primary size, polymer-coated, and hydration layer thickness. The primary size of nuclear magnetic particle is obtained using TME. Hence, the hydrodynamic size (40.1 ± 5.3 nm) is bigger than the primary size (20.0 nm ± 2.5 nm) in our experimental results. Further experiments showed that Gal-CMCS-Fe_3_O_4_-NPs had good magnetic responsiveness in a magnetic field and exhibited strong DNA-binding capabilities in both acidic and alkaline environments, with the strongest binding force at pH 7, which is close to that of the human body. Moreover, the zeta potential which indicated Gal-CMCS-Fe_3_O_4_-NPs could combine with electronegative DNA^38^ was stable across 5 days of observation in water and cell culture media containing DMEM at room temperature. These results suggest that Gal-CMCS-Fe_3_O_4_-NPs are stable at physiological pH, which allows for high transfection efficiencies of Gal-CMCS-Fe_3_O_4_-NPs *in vivo*^39^. Gel electrophoresis and DNA precipitation experiments at different mass ratios showed that the best mass ratio for NPs/DNA binding was 3:1, at which the binding rate of DNA reached 95% (data not shown). Through digestion with DNase I *in vitro*, we observed that Gal-CMCS-Fe_3_O_4_-NPs had an excellent protective effect on DNA. The hemolysis of Gal-CMCS-Fe_3_O_4_-NPs was below 2% . It was reported that up to 5% hemolysis is permissible for biomaterials^30^. These results show that Gal-CMCS-Fe_3_O_4_-NPs have good biocompatibility and provide DNA protection, making them an ideal gene vector.

Cytotoxicity is an important factor influencing the application of gene delivery vectors. At a concentration of 200 μg/ml, Gal-CMCS-Fe_3_O_4_-NPs had a slight toxic effect on liver cells. The transfection of Gal-CMCS-Fe_3_O_4_-NPs had no effect on the shape of normal human liver cells or obvious gross effects in mice. Therefore, Gal-CMCS-Fe_3_O_4_-NPs can feasibly be used as vectors for gene transfection.

We validated the targeting specificity of Gal-CMCS-Fe_3_O_4_-NPs for HCC cells *in vitro* and *in vivo*. Gal-CMCS-Fe_3_O_4_-NPs successfully delivered pcDNA6.2mir-EGFP plasmid into normal human liver cells and human HCC cells without disrupting gene activity. Obvious targeting was not observed for CMCS-Fe_3_O_4_-NPs, as average transfection efficiency was only 20.31% for L02 cells, 17.95% for HepG2 cells, 18.86% for GES-1 cells, 21.01% for U87 cells, and 19.47% for SPCA-1 cells, with no statistical differences between groups (*P* > 0.05). The transfection efficiency of the Gal-CMCS-Fe_3_O_4_-NPs/DNA complex in liver cell lines was higher than that in non-liver cell lines and was also higher than that of the CMCS-Fe_3_O_4_NPs/DNA complex. Before transfection with Gal-CMCS-Fe_3_O_4_NPs/DNA, when a moderate amount of galactose was used to treat HepG2 cells, the transfection efficiency of Gal-CMCS-Fe_3_O_4_NPs/DNA decreased, indicating that the liver-targeting feature of Gal-CMCS-Fe_3_O_4_-NPs was related to its own galactosyl. The biocompatibility of the NPs was improved by modifying the surface with galactose, which allowed for their specific binding with ASGP-Rs on the membrane of liver cells. This binding enabled delivery of the NPs/DNA compound into the cells, thus increasing the transfection efficiency^40^. We also showed that the presence of an external magnetic field improved the transfection efficiency of Gal-CMCS-Fe_3_O_4_-NPs, perhaps by controlling the direction of travel of the magnetic NPs. With the aid of an external magnetic field, NPs/DNA compound can quickly concentrate and attach to the surface of single-layer cultivated cells, thereby enhancing the speed and strength of contact between the NPs/DNA compound and cells^41^. After injection of Gal-CMCS-Fe_3_O_4_-NPs/DNA into the tail vein of mice, green fluorescence was detected in liver and orthotopically transplanted HCC tissue but not in other organs such as the heart, spleen, lungs, and kidneys. Furthermore, no fluorescence was observed in organs of mice treated with CMCS-Fe_3_O_4_-NPs or naked DNA (data not shown). These results suggest that Gal-CMCS-Fe_3_O_4_-NPs may be a good DNA transfection vector for targeting the liver *in vivo*.

Although the diagnosis and treatment of HCC has greatly improved over the past two decades, transarterial chemoembolization or chemotherapy still plays an important role in its treatment^42^. Because most HCC patients have a medical history of posthepatitic cirrhosis and hepatic insufficiency, the appropriate dose, intensity, and mode of chemotherapy is difficult to determine^43^. Moreover, the lack of tumor suppressor gene expression in HCC cells can lead to defects in apoptosis-related signal transduction pathways and promote tolerance to chemotherapy^44^,^45^, thus limiting the efficacy of chemotherapy for HCC. Re-expressing an inactive tumor suppressor gene through a transgene vector can restore apoptosis in tumor cells, providing a new strategy for enhancing the sensitivity of HCC cells to chemotherapy. This would allow for reduced dosage of chemotherapy drugs and reduced toxic side effects^13^.

In this study, we successfully validated the antitumor efficacy of Gal-CMCS-Fe_3_O_4_-NPs/RASSF1A compound by observing the expression of RASSF1A protein in orthotopically transplanted HCC tissue and the inhibition of tumor growth in mice. Furthermore, the presence of an external magnetic field increased RASSF1A expression, slowed the growth of tumors, and enhanced the anti-proliferative effect of Gal-CMCS-Fe_3_O_4_-NPs/RASSF1A compound. Moreover, the increased expression of RASSF1A was associated with greater MMC-induced apoptosis of HCC cells, indicating that by inducing the apoptosis of targeted cells, the expression of RASSF1A can enhance the sensitivity of HCC cells to chemotherapy. To explore the mechanism by which RASSF1A regulates apoptosis, we used western blot analysis to measure changes in caspase-3, p53, p21, and bax levels in HCC tissue. The level of activated caspase3 in HCC tissue increased as the expression of RASSF1A increased. Therefore, in addition to the effect of MMC on HCC cells, the RASSF1A gene may activate caspase-3 through specific signaling pathways, thereby promoting apoptosis of HCC cells and increasing the sensitivity of HCC cells to chemotherapy^46^.

In conclusion, Gal-CMCS-Fe_3_O_4_-NPs are a new vector for the efficient gene transfection of HCC cells that is safe, effective, and feasible. Gal-CMCS-Fe_3_O_4_-NPs serve a dual targeting function; that is, their transfection efficiency can be improved with the aid of an external magnetic field and they can deliver a gene specifically to HCC cells through ASGP-Rs. Transfection of HCC cells with the RASSF1A gene using Gal-CMCS-Fe_3_O_4_-NPs inhibited the growth of tumors and increased the sensitivity of HCC cells to chemotherapy, suggesting the importance of RASSF1A for HCC gene therapy.

## Methods

### Materials and reagents

CMCS was provided by the Institute of Neuroscience, Nantong University. FeCl_3_, FeCl_2_, HCl, NaOH, lactose, and cyano sodium borohydride were domestically obtained. pcDNA3.1(+)RASSF1A was a gift from Dr. Gerd P. Pfeifer^47^. The pcDNA6.2mir-EGFP plasmid was purchased from Invitrogen (Carlsbad, CA, USA). The human HCC cell line HepG2, human normal liver cell line L02, human gastric mucosa cell line GES-1, human glioma cell line U87, and human lung adenocarcinoma cell line SPCA-1 were purchased from the cell bank of the Chinese Academy of Sciences (Shanghai, China).

### Synthesis of Gal-CMCS-Fe_3_O_4_-NPs

Under the protection of nitrogen, FeCl_3_ (10.8 g, 0.067 mol) and FeCl_2_ (4 g, 0.031 mol) were added to 50 ml HCl (1.1 mol/l) and filtered through a 0.22-μm filter to remove bacterium. The solution (25 ml) was quickly poured into 250 ml NaOH (1.5 mol/l) and agitated for 1 h at 80 °C. The combined solution was then poured into a 500-ml beaker attached to a permanent magnet. Supernatants were discarded after the black material had completely precipitated. Double-distilled water was used for flushing until sedimentation no longer occurred. When the pH was approximately 8, Fe_3_O_4_-NPs were isolatd following cooling and drying.

The CMCS-Fe_3_O_4_-NPs was prepared in accordance with the literature with minor modification^48^. Briefly, under the protection of nitrogen. The separated Fe_3_O_4_-NPs (140 mg, 0.6 mmol) were re-suspended in 40 ml PBS with 120 mg EDC (1-ethyl-3-(3-dimethylaminopropyl) and 120 mg NHS (N-hydroxysuccinimide, Pierce, Rockford, USA), then 280 mg CMCS was added immediately. The solution was dispersed for 2 h at room temperature with ultrasonic waves. A permanent magnet was used to isolate the magnetic compound that was subsequently washed twice with with ethanol. Double-distilled water was added to a constant volume of 60 ml, and a colloid solution of CMCS-Fe_3_O_4_-NPs was obtained and evenly dispersed with ultrasonic waves at 37 °C. Lactose (336 mg, 3.7 mmol) and sodium cyanoborohydride (168 mg, 2.7 mmol) were slowly added, and the solution was agitated for 1 h at 37 °C. The magnetic compound was isolated with a permanent magnet, washed twice with ethanol (30 ml), freeze-dried in a vacuum, and preserved for later use.

### Characterization of Gal-CMCS-Fe_3_O_4_-NPs

Size, morphology, and electron diffraction of Gal-CMCS-Fe_3_O_4_-NPs were observed using a TEM (JEOL JEM-2010, Tokyo, Japan) operated at 200 kV. The size distribution and surface charge of Gal-CMCS-Fe_3_O_4_-NPs were measured as the zeta potential using a Nicomp 380 ZLS instrument (PSS, Santa Barbara, CA, USA). The surface chemistry of Gal-CMCS-Fe_3_O_4_-NPs was studied using a Fourier transform infrared spectrometer (AVATAR-370, Thermo, Madison, WI, USA) with KBr as a diluting agent and scanned against a blank KBr pellet background. A thermogravimetric analyzer (Shimadzu TGA-50 Analyzer, Tokyo, Japan) was used to perform thermal analyses. The saturation magnetization for Gal-CMCS-Fe_3_O_4_-NPs was done using Lake Shore 7407 vibrating sample magnetometer (Lake Shore Cryotronics, Westerville, OH, USA).

### Lectin-induced aggregation

Gal-CMCS-Fe_3_O_4_-NPs and CMCS-Fe_3_O_4_-NPs were separately incubated with RCA120 (0.5 mg/mL) in phosphate-buffered saline (90 μl) before adding galactose(10 μl, 100 mM). The turbidity of 4 wells was monitored every minute using an enzyme-linked immunosorbent assay plate reader (Bio-Tek Elx 800, Winooski, VT, USA) at 450 nm. Every experiment repeated three times.

### Agarose gel retardation assay

The reporter pcDNA6.2mir-EGFP plasmid was purified using an EndoFree Plasmid Mega Kit (Qiagen Co. Ltd., Shanghai, China) according to the manufacturer’s instructions. Gal-CMCS-Fe_3_O_4_-NPs and pcDNA6.2mir-EGFP were mixed at mass ratios of 3:1 in a 50-μl reaction system with pH values adjusted to 5, 7, or 9, or at mass ratios of 0.5:1, 1:1, 2:1, 3:1, or 4:1 with the pH adjusted to 7. After 1 h at room temperature, the reaction products were removed for electrophoresis on 0.5% agarose gel at 80 V for 2 h. Gels were imaged using a gel imaging system. At a pH of 7, Gal-CMCS-Fe_3_O_4_-NPs and pcDNA6.2mir-EGFP plasmid were mixed at the best mass mixture ratio. After 1 h at room temperature, 0.5 U DNase I or fresh mouse serum was added to the mixtures in a water bath for 1 h at 37 °C. The compounds were separated on 0.5% agarose gels.

### Hemolysis assay

Hemolysis of red blood cells (RBCs) was examined as previously described^30^. Briefly, 1.5 mL of fresh rat RBCs were harvested by centrifuging at 1500 rpm for 10 mins. The resultant RBC suspension was washed three times with NS. Finally, the RBCs were resuspended in NS to a concentration of 2% (v:v). Then, 0.7 ml of diluted 2% RBC suspensions were added to varying concentrations of 0.1 mL of Gal-CMCS-Fe3O4-NPs solutions in NS (25, 50, 100, 150, 200, 250, and 300 μg/ml). The resultant mixtures were incubated at 37 °C for 2 h and then centrifuged at 1500 rpm for 5 mins. The absorbance of the supernatant was measured for release of hemoglobin at 545 nm. The percentage of hemolysis was calculated as follows: % hemolysis = (OD_t_-OD_n_)/(OD_p_-OD_n_) × 100. Where, OD_t_, OD_n_, and OD_p_ are the absorbance values of the test sample, negative control (NS), and positive control (water), respectively. All the hemolysis experiments were performed in triplicate.

### Cytotoxicity assessment *in vitro*

L02 cells were seeded at a density of 5 × 10^3^ cells/well in a 96-well microtiter plate and were cultured in DMEM with Gal-CMCS-Fe_3_O_4_-NPs at 0(as control), 10, 25, 50, 100, 150, 200, 250, or 500 μg/ml. After 72 h, cellular morphology was observed using an inverted microscope, and the growth of cells from four wells was assessed using a Cell Counting Kit-8 (CCK-8, Beyotime Institute of Biotechnology, Jiangsu, China) according to the manufacturer’s instructions. Cell viability (%) was calculated as the mean optical density of treated wells/mean optical density of control wells ×100.

### *In vitro* transfection

Galactose (1 ml, 100 mM) were added 15 min before transfection. HepG2 cells were co-incubated with Gal-CMCS-Fe_3_O_4_-NPs prepared with pcDNA6.2mir-EGFP plasmid (DNA content 2 μg) at an N/P ratio of 3:1. After a 72-h co-incubation, cells were fixed in 4% paraformaldehyde for 30 min followed by nuclear staining with 4′,6-diamidino-2-phenylindole (DAPI; Beyotime Biotech Inc.). An inverted fluorescence microscope was used to observe the transfected cells, and flow cytometry was used to determine the percentage of transfected cells. Every experiment repeated three times.

### Cytotoxicity assessment *in vivo*

Gal-CMCS-Fe_3_O_4_-NPs (90 μg, 100 μl) and the same volume of NS as control were were injected into the tail vein of BALB/C nude mice(*n* = 50). At 1, 2, 3, 7, or 14 days after injection, 5 mice of each group were sacrificed, and blood serum samples were collected , and levels of aspartate transaminase (AST), alanine transaminase (ALT), and total bilirubin (T-BIL) were measured. The influence of NPs on the morphology of various organs and tissues was also observed at the 14th day after injection.

### Orthotopic transplantation tumor model of HCC

HepG2 cells (1 × 10^6^) were injected subcutaneously into the flanks of 4-week-old male BALB/C athymic nude mice. Tumorectomies were performed when the subcutaneous tumors grew to a diameter of 1 cm. A small piece (approximately 1–2 mm^3^) of prepared fresh tumor tissue was implanted into the capsule of the liver lobe in nude mice at an angle of 20°. Absorbable sutures (7–0) were used for local stiffening. All of the animal protocols were approved by the Animal Care and Use Committee of Nantong University and the Jiangsu Province Animal Care Ethics Committee (Approval ID: SYXK (SU) 2007-0021), and the methods were carried out in accordance with the approved guidelines.

### *In vivo* transfection of Gal-CMCS-Fe_3_O_4_-NPs

Fourteen days after orthotopic HCC tumor transplantation, Gal-CMCS-Fe_3_O_4_-NPs or CMCS-Fe_3_O_4_-NPs (90 μg) and pcDNA6.2mir-EGFP plasmid (30 μg) were mixed and injected into the tail vein of 5 nude mice respectively. Mice were sacrificed 72 h after injection and livers, pieces of tumor, spleen, kidneys, heart, and lungs were removed and frozen. Tissue sections (6 μm) were cut on a cryostat and nuclear staining was performed with DAPI. Fluorescent microscopy was performed to visualize GFP expression. Transfection efficiency was calculated as percentage of cells expressing GFP by counting the number of the cells that display or do not display GFP signals in five areas (the upper left, the upper right, the bottom left, the bottom right, and the center) under four randomly chosen microscopic visions^49^.

### *In vivo* therapeutic effect of NPs in an orthotopic transplantation model of HCC

Fourteen days after orthotopic HCC tumor transplantation, nude mice (*n* = 90) were randomly divided into five groups, and there were 16 mice in each group. Mice in group ‘a’ received an injection of Gal-CMCS-Fe_3_O_4_-NPs/pcDNA3.1(+)RASSF1A compound through the caudal vein and an intraperitoneal injection of MMC (Kyowa Hakko Kogyo Co. Ltd., Japan). A small permanent magnet was affixed to the upper abdomen of nude mouse(Fig. 7A), so that a magnetic field was applied to the tumor area. Mice in group ‘b’ received an injection of Gal-CMCS-Fe_3_O_4_-NPs/pcDNA3.1(+)RASSF1A compound through the caudal vein and an intraperitoneal injection of MMC. A magnetic field was not applied to the tumor area. Mice in group ‘c’ received an injection of Gal-CMCS-Fe_3_O_4_-NPs/pcDNA3.1(+)RASSF1A compound through the caudal vein and an intraperitoneal injection of NS. A magnetic field was applied to the tumor area. Mice in group ‘d’ received an injection of Gal-CMCS-Fe_3_O_4_NPs/pcDNA3.1(+) compound through the caudal vein and an intraperitoneal injection of MMC. A magnetic field was applied to the tumor area. Mice in group ‘e’ (control group) received an injection of Gal-CMCS-Fe_3_O_4_-NPs/pcDNA3.1(+) compound through the caudal vein and an intraperitoneal injection of NS. A magnetic field was applied to the tumor area. Gal-CMCS-Fe_3_O_4_-NPs or CMCS-Fe_3_O_4_-NPs (90 μg) and pcDNA3.1(+)RASSF1A plasmid (30 μg) were mixed and injected into the tail vein of nude mice. Intraperitoneal injection of MMC (0.7 mg/kg) was performed once. Two weeks later, tumors were dissected and weighed, and tumor volume (mm^3^) was calculated as 0.5 × L (length, mm) × W^2^ (width, mm^2^).

### TUNEL staining

Apoptotic cells in tumor tissue were detected using TUNEL staining of serial 4-μm sections cut from paraffin-embedded tumor tissues using an *in situ* cell death detection kit (Roche, Mannheim, Germany). Each group randomly selected 8 nude mice from 16 mice. Staining was performed according to the manufacturer’s instructions. The proportion of apoptotic cells in each group was measured in five areas (the upper left, the upper right, the bottom left, the bottom right, and the center) under four randomly chosen microscopic visions.

### Western blot

Proteins were extracted from the tissues using RIPA lysis buffer (Beyotime) containing phosphatase inhibitor (100:1). Then the lysates were centrifuged at 14 000 rpm for 20 minutes. Western blot analysis was performed as previously described^50^. Briefly, the protein was transferred into PVDF membrane after separating from 10% SDS-PAGE. Primary antibodies used included anti-RASSF1A (eBioscience, San Diego, CA, USA), anti-caspase-3, anti-p53, anti-bax, and anti-p21 (Santa Cruz Biotechnology, CA, USA). Antibodies were diluted according to the manufacturers’ instructions.

### Statistical analysis

Quantitative data are shown as mean ± standard error of the mean of at least three independent experiments. Statistical analysis was performed using t-tests with SPSS/Win13.0 software (SPSS, Inc., Chicago, IL, USA). A P-value < 0.05 was considered statistically significant.

## Additional Information

**How to cite this article**: Xue, W.-J. *et al*. Asialoglycoprotein receptor-magnetic dual targeting nanoparticles for delivery of RASSF1A to hepatocellular carcinoma. *Sci. Rep*. **6**, 22149; doi: 10.1038/srep22149 (2016).
